# Fractures of the Cervical Vertebræ

**Published:** 1829-01

**Authors:** 


### FRACTURES OF THE CERVICAL VERTEBRÆ.

Case I.—James Halford, admitted into St. Bartholomew's Hospital, on the evening of November 10th, about eight o'clock, with complete paralysis of upper and lower extremities, consequent to injury of the cervical vertebræ, occasioned by a fall from the top of a loaded waggon. When admitted, had not the power of raising his head, had lost all sensation and power of motion, excepting that he was able to turn his head from side to side when in bed; complained of great pain in the back of the neck; respiration slow and oppressed, performed by the diaphragm only, the intercostals being completely paralysed; pulse about sixty, somewhat full; partial priapisms.

On examining the back of the neck, the spinous process of the fifth cervical vertebra was found to be displaced, and more prominent than usual. From this and the attending symptoms, it was concluded that there was fracture probably extending through the bodies of the cervical vertebræ. During the night the priapisms became more frequent, and towards morning a quantity of flatus had collected in the abdomen; nothing had passed per rectum, and an enema was ordered. With the view of exciting the muscular action of the intestines through the nervous influence, a stream of galvanism was directed from the back through to the rectum: this at first had the effect of somewhat lessening the distention, but the tympanitis soon increased, and continued to extend, though the galvanism was kept up for upwards of half an hour, varying its direction: occasionally it was applied with less power through the œsophagus to the rectum. The patient was bled to about six ounces, when he became sick and faint; a drop of croton oil was given in two doses, but without any effect; the water was drawn off twice, the smell of which was slightly ammoniacal. He gradually sunk from the oppression, continually turning his head from side to side, and died at six p.m.

On dissection, it was ascertained that dislocation had taken place between the fourth and fifth cervical vertebræ. The ligamentum subflavum was torn throughout, and the apex of the fourth spinous process lay in close contact with the basis of that of the fifth. The antero-posterior diameter of the vertebral canal was lessened by this displacement nearly one-half; the spinal marrow was soft and pulpy, and blood was effused in its substance; its membranes were entire; some blood was effused in the canal between the bones and the membranes.

Case II.—November 11th, J. Taylor, about sixty, was admitted into the same hospital in a state of complete paralysis of the whole body, caused by a fall from a height of about fourteen feet. There was a depression of one or more of the lower cervical vertebræ; he complained of pain upon pressing the injured part, and also of pain in the right arm just below the elbow, and at the outside; he asked if the skin was rubbed off from that part; he had no priapism; pulse small and rather slow; breathing laborious; perfectly sensible.

12th.—Symptoms much the same; priapism took place during the day; has had no evacuation since the accident, though two injections have been given. About a pint of urine was drawn off in the morning, and nearly half a pint more in the evening. Pulse same as last night. Respiration became more laborious, and he died about three o'clock in the morning.

*Dissection.*—The situation of the displacement was between the sixth and seventh cervical vertebræ, the articular processes were broken, and the ligamentum subflavum and fibro-cartilage torn; the spinal cord was crushed, and blood effused in small spots in its substance; the lungs were loaded with blood, with a little mucus in the air-cells.

